# 2019‒2020 Australian bushfire air particulate pollution and impact on the South Pacific Ocean

**DOI:** 10.1038/s41598-021-91547-y

**Published:** 2021-06-10

**Authors:** Mengyu Li, Fang Shen, Xuerong Sun

**Affiliations:** State Key Laboratory of Estuarine and Coastal Research, East China Normal University, Shanghai, 200241 China

**Keywords:** Environmental sciences, Natural hazards, Ocean sciences

## Abstract

During late 2019 and early 2020, Australia experienced one of the most active bushfire seasons that advected large emissions over the adjacent ocean. Herein, we present a comprehensive research on mixed atmospheric aerosol particulate pollution emitted by wildfires in the atmosphere and the ocean. Based on a wide range of physical and biochemical data, including the Aerosol Robotic Network, multi-satellite observations, and Argo floats, we investigated the spatio-temporal variations and mixed compositions of aerosol particles, deposition in the coastal waters of eastern Australia and the South Pacific Ocean, and biogeochemical responses in the water column. Four types of wildfire-derived mixed particles were classified by using the optical properties of aerosols into four types, including the background aerosols, mineral dust, wildfire smoke particles, and residual smoke. The coarse particles accounted for more than 60% of the mineral dust on 22 November 2019 in the Tasman Sea; afterwards, during the wildfire smoke episode from December 2019 to January 2020, the particles affected large areas of the atmosphere such as eastern Australia, the South Pacific Ocean, and South America. The maximum value of the aerosol optical depth reached 2.74, and the proportion of fine particles accounted for 98.9% in the smoke episode. Mineral dust and smoke particles from the fire emissions changed the particle composition in the surface ocean. Particle deposition accounted for increases in chlorophyll-a concentration (Chl*a*) standardized anomaly up to maximum of 23.3 with a lag time of less than 8 days. In the vertical direction, float observations showed the impact of exogenous particles on the water column could up to 64.7 m deep, resulting in Chl*a* of 1.85 mg/m^3^. The high Chl*a* lasted for a minimum period of two months until it returned to normal level.

## Introduction

Massive bushfires fuelled by record-breaking high temperatures and months of extreme drought raged in eastern Australia during the summer of 2019–2020^1^. The fires destroyed 3113 houses, claimed 33 lives, and generated an estimated economic impact of up to 20 billion dollars^2^. The pyrocumulonimbus generated by the wildfire lifted enormous amounts of particles into the atmosphere, significantly impacting air quality, visibility^3,4^, and human health^5–7^. Field measurements suggest that intense bushfires could provide a source of other nutrients, including nitrogen and phosphorus, and especially iron, which may be associated with biomass-burning smoke particles^8–11^ and dust particles entrained by pyro-convection^6,12^. The mixing of dust and smoke particles can significantly increase aerosol loading and modify the optical properties of the background atmosphere^13^.

Furthermore, the entry of nutrients-containing particles into the marine environment may potentially alter ocean primary production, thereby exerting significant influence on marine ecosystems^14,15^, controlling the exchange of carbon to the deep ocean via bio-pumps^16,17^, and even driving climate change^14,15^. Previous studies have shown that iron-containing particle deposition can induce phytoplankton growth because of the addition of nutrients to oligotrophic regions^18–21^. Compared to mineral dust particles, the results of a number of studies pointed toward an elevated solubility of iron in aerosols produced by the combustion of fossil fuels and biomass, which can potentially provide more bioavailable Fe to the surface ocean^9,22–24^. Atmospheric mineral dust contained approximately 3% Fe by mass and a tiny fraction of soluble Fe, which is usually considered Fe (II) (although other forms of Fe may also be bioavailable)^25,26^. Bioavailable Fe derived from direct emissions from combustion sources and processes by anthropogenic atmospheric pollutants in mineral dust play a vital role in ocean fertilisation over various surface ocean areas, where primary production and nitrogen fixation are limited by Fe scarcity^9,27,28^. Thus, biomass-burning particles may play a significant role in regional ocean fertilisation.

The South Pacific Ocean is isolated from most continental sources, has low chlorophyll-a concentrations (Chl*a*, see Table 1 for a full list of symbols, terms, definitions, and units), and thus represents an end-member of oceanic hyper-oligotrophic conditions^29^. Due to nutrient limitations, oligotrophic areas are likely to respond significantly to changes in atmospheric deposition^25^. Analyses of Australian continental waters have shown that ocean primary productivity responded to dust deposition in the coastal ocean^30,31^. However, comprehensive studies on mixed particulate pollution in both the atmosphere and the ocean are relatively scarce^10,25^. The hypothesis is that bushfire-derived particulate air pollution may have a significant impact on regional oligotrophic ocean fertilisation and primary productivity.

In this study, we utilised multi-sourced and multipurpose datasets to present a detailed analysis of the 2019‒2020 Australian air particulate pollution and its impact on coastal and open ocean waters. Detailed compositional analyses of atmospheric pollutants and spatio-temporal variations from fire combustion were conducted. The impacts of particle deposition on the surface ocean were evaluated and found to change the water composition. Finally, we investigated the ocean biological responses in the water column to different atmospheric particulate pollution types to evaluate their impact on marine ecology. This study highlights the significance of bushfire-derived dust in the marine carbon cycle in the context of climate change.

**Table 1:** Symbols, terms, definitions and units.

Symbols and abbreviations	Description
*δ*	Residual scattering coefficient
Δ*X*	Standardized anomalies of variables, *X* can be Chl*a*, *b*_*bp_443*_, *a*_*ph_443*_, UVAI, or SST
Δ*σ*_*θ*_	Change in potential density, in kg/m^3^
*α* (443), *β* (443)	Regression coefficients at 443 nm for *δ*
AE	Angstrom exponent
AERONET	Aerosol robotic network
AOD_440_	Aerosol optical depth at 440 nm from AERONET site, dimensionless
*a*_*ph_*443_	Absorption coefficient of phytoplankton at 443 nm on the ocean surface, in m^-1^
Argo	An international program that uses profiling floats to observe oceans
*b*_*bp*_443_	Particulate backscattering coefficients at 443 nm on the ocean surface from MODIS, in m^-1^
*b*_*bp*_700_	Particulate backscattering coefficients at 700 nm in the water columns from Argo floats, in m^-1^
BGC-Argo	Biogeochemical Argo
Chl*a*	Chlorophyll-a concentrations, in mg/m^3^
*dV(r)/dlnr*	Volume particle size distribution in air, in µm^3^/µm^2^
GAMSSA	Global Australian Multi-Sensor SST Analysis
GHRSST	The Group for High Resolution Sea Surface Temperature
GIOP	Generalised Inherent Optical Property
MLD	Mixed Layer Depth
MODIS	Moderate Resolution Imaging Spectroradiometer
NASA	National Aeronautics and Space Administration
OMI	Ozone Monitoring Instrument (OMI) on board the Aura satellite
*r*	Equivalent spherical radius of air particulate, in µm
*r*_VF_	Median radius for fine particles
SDA	Spectral Deconvolution Algorithm
SOCCOM	Southern Ocean Carbon and Climate Observations and Modeling project
SST	Sea Surface Temperature
TROPOMI	Tropospheric Monitoring Instrument
UVAI	Ultraviolet Aerosol Index from OMI
V_C_	Volume concentrations of coarse particles
V_F_	Volume concentrations of fine particles

## Results

### Bushfire-derived air particulate pollution

The MODIS active fire products showed that the 2019–2020 Australian bushfire season was unprecedented compared to years of moderate fire points (e.g., 2012–2013 bushfire season) and years of low fire activities (e.g., 2014–2015 bushfire season). Table 2 shows the numbers of fire points for each typical bushfire season compared to climatology. It can be seen that the overall anomalies of active fire points for the 2019–2020 bushfire season were higher compared to the typical years. The number of fire point anomalies for the 2019–2020 bushfire season reached a maximum in December 2019 exceeding the 20-year average by over 1000 counts. And the 2019–2020 bushfires in Australia unleashed 8500.3 Gg and 17,319.2 Gg carbon into the atmosphere on 9 November 2019, and 5 January 2020 respectively (Supplementary Fig. 1).

**Table 2:** Monthly average anomalies active fire points for the typical months in Australia.

Bushfire season	November	December	January	February
2012–13 (moderate bushfire)	784.60	400.01	158.89	19.39
2014–15 (normal year)	− 26.57	− 71.54	− 115.79	4.46
2019–20 (this study)	209.90	1017.14	438.40	31.25

**Figure 1:**
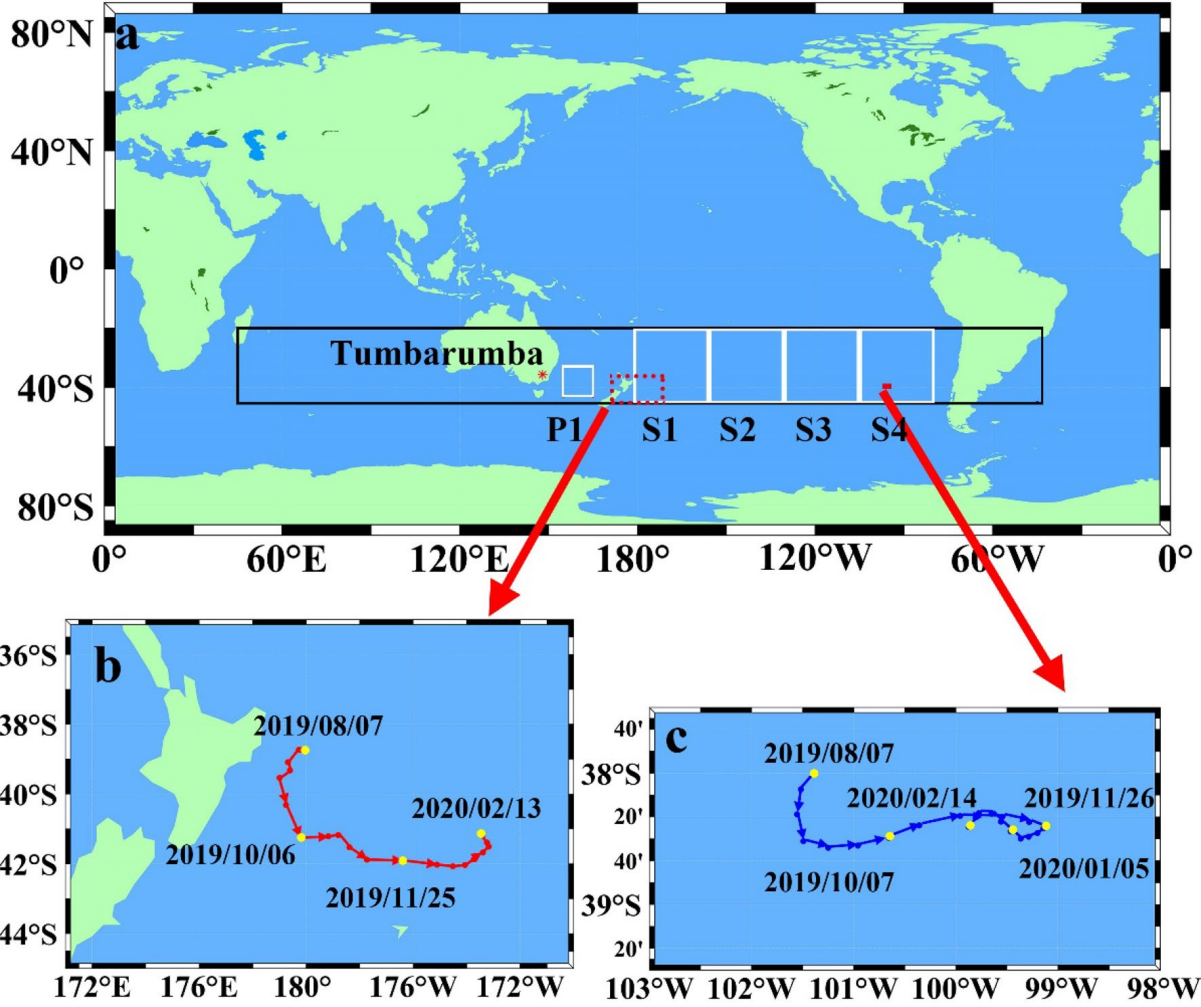
(**a**) Map of areas of interest (generated using MATLAB 9.9.0.1570001 R2020b Update 4, https://www.mathworks.com/products/matlab.html). The black rectangle (20°S–45°S, 45°E–45°W) indicates the Hovmoller latitude-averaged plot in Fig. 2a. The red star represents the location of the AERONET site (Tumbarumba, Australia). The white boxes represent P1 (33°S–43°S, 155°E–165°E) and P2 (20°S–45°S, 180°–80°W), which contains 4 subparts of the same size (i.e., S1–S4). (**b**) Position and trajectory of No. 5905108 BGC-Argo float. (**c**) Position and trajectory of No. 5904843 BGC-Argo float.

**Figure 2:**
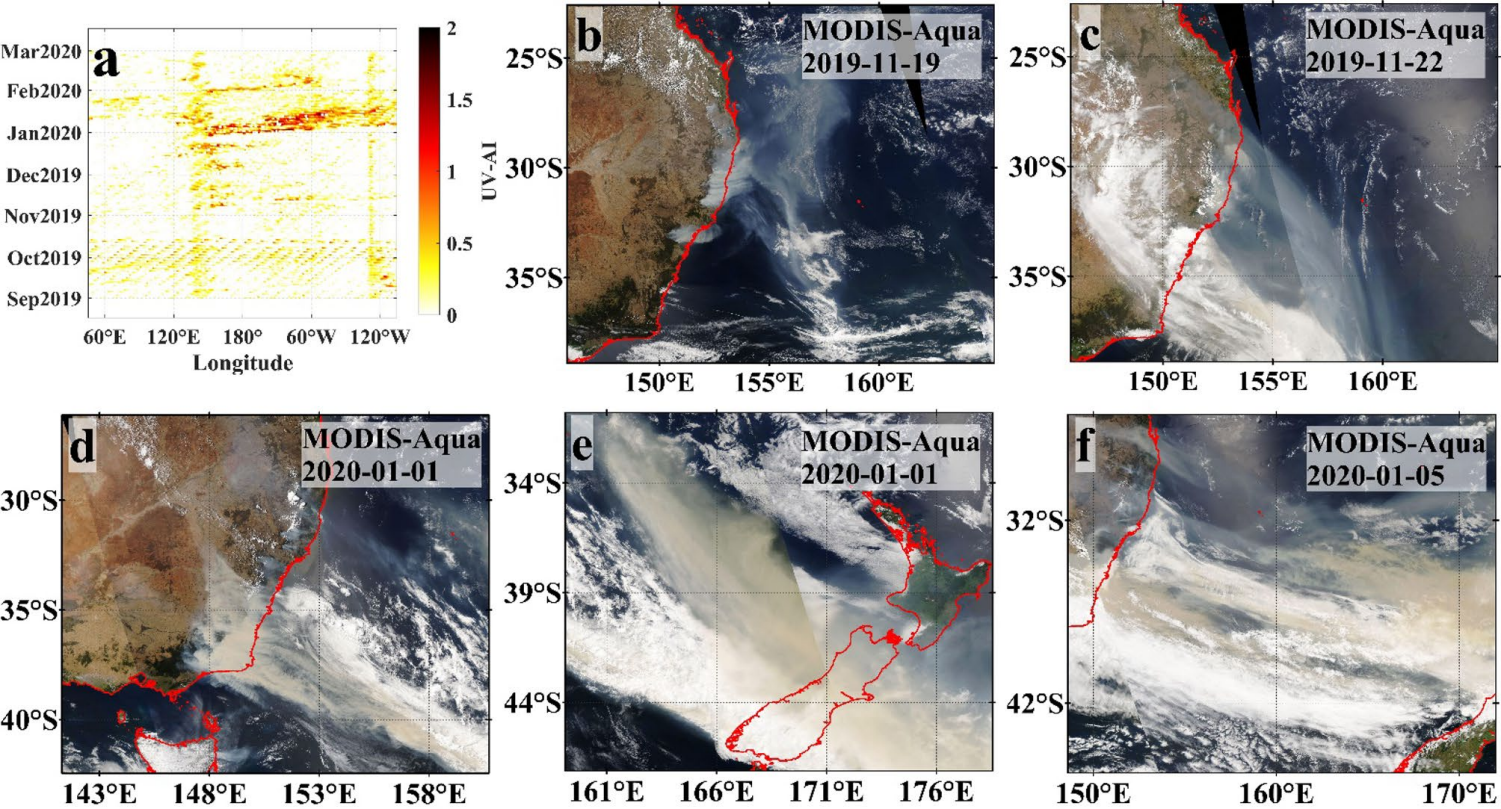
The Hovmoller latitude-averaged UVAI in the range of the black box in Fig. 1. MODIS Aqua reflectance images (true colour) of the atmospheric pollution observed by MODIS (**b**,**c**) on 19 and 22 November 2019 and (**d–f**) heavy particulate plumes covering the Tasman Sea and beyond. The coastline is shown in red lines. MODIS Aqua images were downloaded from the NASA Worldview website (https://worldview.earthdata.nasa.gov).

Satellite images provide detailed information on pollution. In addition to the small number of particles released in September in New South Wales and Queensland, satellites also recorded a large number of particles released by bushfires in the coastal area of southeastern Australia in November 2019 and January 2020. A Hovmoller latitude-averaged graph (Fig. 2a, the study area is showed in Fig. 1, 20°S–45°S, 45°E–45°W) Fig. 1 was plotted for ultraviolet aerosol index (UVAI) over an area from 20°S, 45°E to 45°S, 45°W. The chart shows that fire emissions primarily affected the atmosphere on the east coast of Australia in late November 2019 and the South Pacific Ocean from January to early February 2020. Figure 2b,c show the fire-driven air particulate pollution process observed by MODIS-Aqua on 19 November 2019. The particle plumes extended over the southeastern coast sea, following the prevalent wind. Detailed trends of the emissions covering the southeast coast of Australia and the Tasman Sea from 6 November to 14 December 2019 are provided in Supplementary Figure 2. Figure 2d‒f show that on 1 and 5 January 2020, the uncontrolled bushfire emissions in Victoria dramatically increased, spread to the coast of southeastern Australia, and covered the Tasman Sea and New Zealand. The satellite images (Supplementary Fig. 3) indicated that pollution was transported from the bushfire regions in southeastern Australia to the South America from 26 December 2019 (Australia local date) to 20 January 2020. By mid-January 2020, emissions were carried across the Southeast Pacific to Chile and Argentina.

### Temporal variation of aerosol types from AERONET

**Figure 3:**
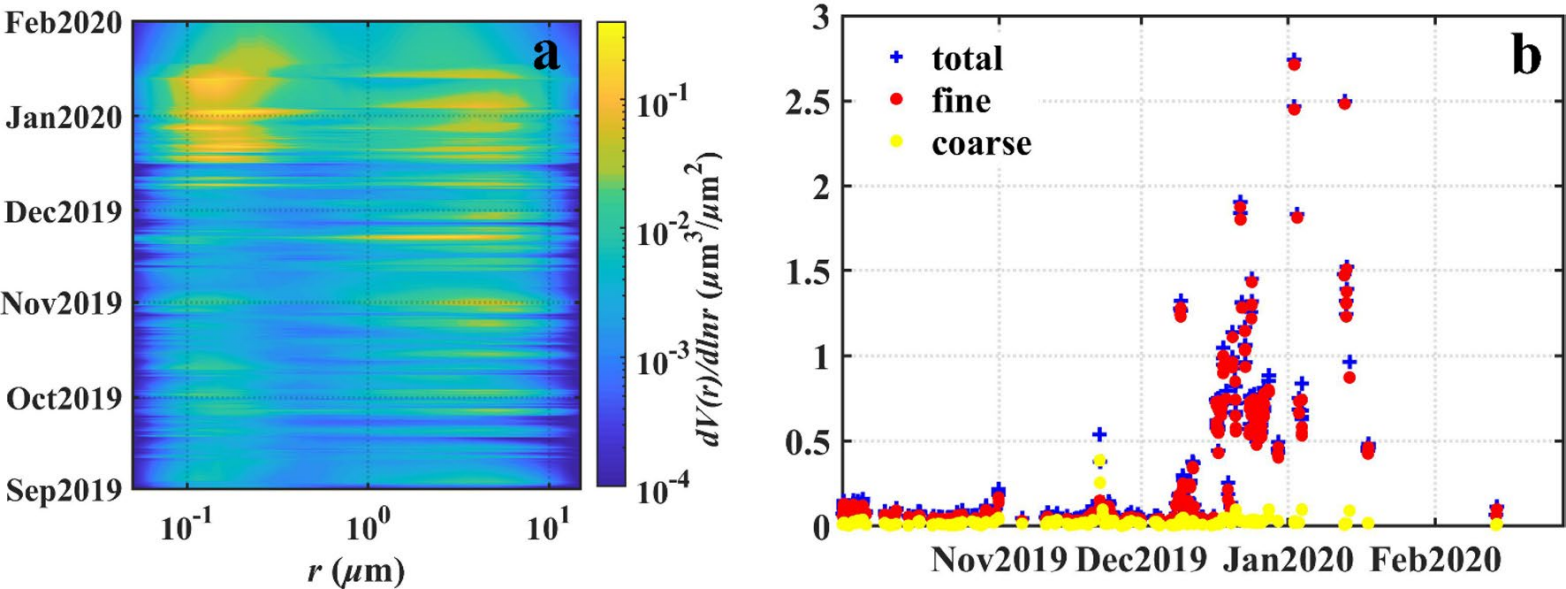
(**a**) Aerosol particle size distribution from September 2019 to February 2020 at the Tumbarumba site. (**b**) Daily SDA retrievals of the total, fine, and coarse mode AOD_440_ values from October 2019 to February 2020 at the Tumbarumba site.

Atmospheric fine, coarse, and total aerosol optical depth (AOD) and the particle size distribution data of the station (marked with the red star in Fig. 1) are illustrated in Fig. 3a,b. Coarse particulate emissions on 22 November 2019 temporarily reached a total AOD at 440 nm (AOD_440_) value of 0.54, with a coarse particle contribution of more than 60%, compared to 14.2% in the median during the non-biomass burning episode. By mid-December 2019, the dominant particle size changed from coarse to fine. The total AOD_440_ increased significantly, reaching a maximum of 2.74 on 2 January 2020, while the proportion of fine-mode particles accounting for 98.9%.

**Figure 4:**
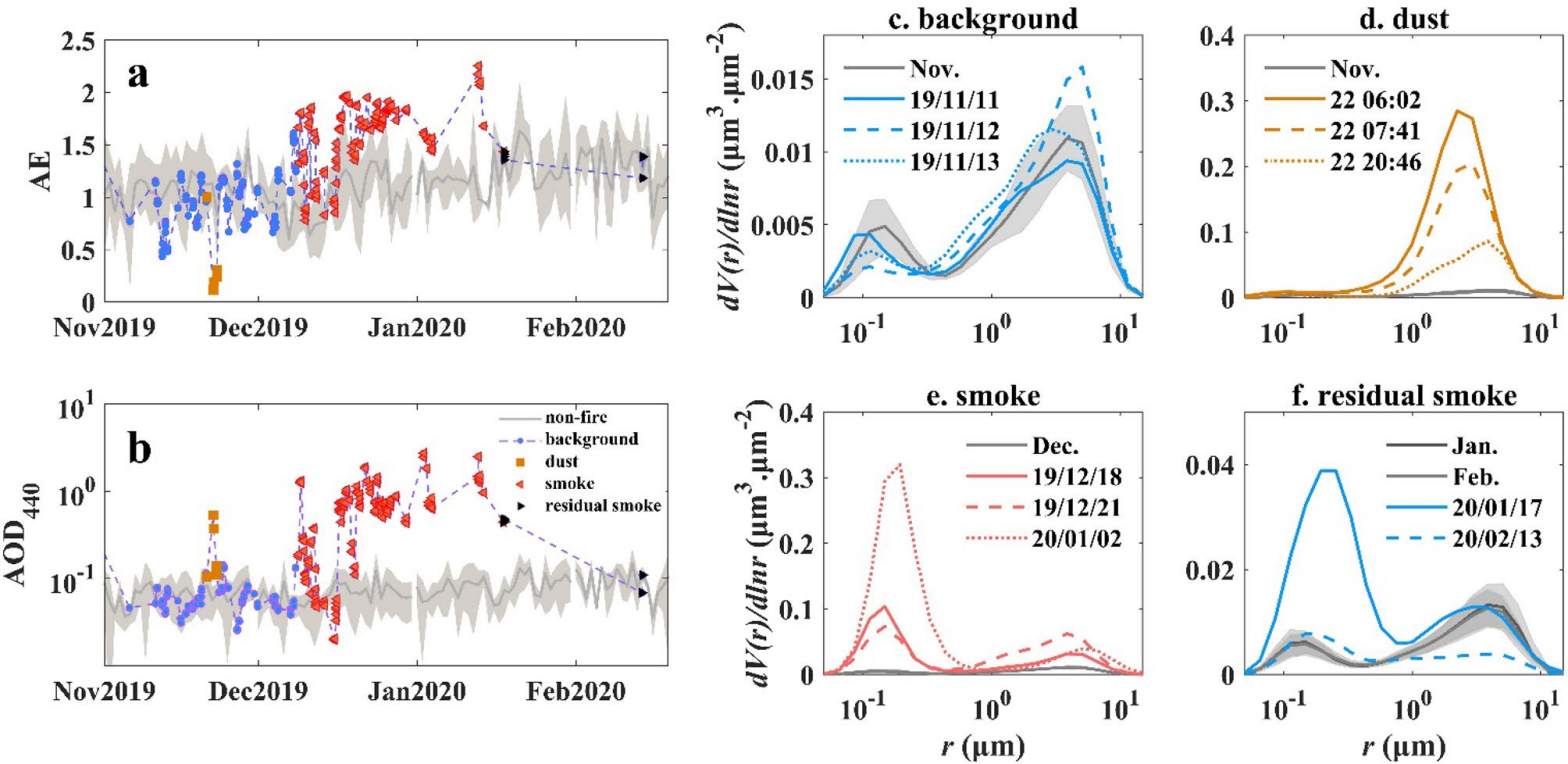
AERONET observations from November 2019 to late February 2020: AE (**a**) and total AOD_440_ (**b**) and aerosol particles size distributions for the aerosol types of the study time: background (**c**), dust (**d**), smoke (**e**), and residual smoke (**f**) according to the method in Gómez-Amo et al. (2017).

Figure 4 shows a detailed, colour-coded classification of the aerosol type identification for each observation. According to the identification method described in “Aerosol types identification”, both AOD_440_ and angstrom exponent (AE) were used to classify aerosol types. When AOD increased significantly, aerosol types were classified according to AE value. Aerosol types were classified by the values of AE in the presence of a significant increase in AOD. Compared with the usual situation, AE in the dust period was lower, while the AOD was slightly higher than that in previous years. During the smoke period, both AE and AOD increased significantly. We found four types of aerosols according to the dominant particles based on AOD and AE observations: (1) background aerosols before 22 November 2019 (blue in Fig. 4); (2) dust particulate pollution on 22 November 2019 (red-brown); (3) wildfire smoke from 8 December 2019 to 13 January 2020 (red); and (4) residual subsidence after 17 January 2020 (black). AOD_440_ < 0.1 and AE ~ 1 indicated the local background aerosols before the air particulate pollution event. AOD_440_ > 0.15 and AE rapidly decreasing to less than 0.5 suggested a dust episode (Fig. 4a,b). Mineral dust particles primarily influence the coarse mode of the size distribution by enhancing AOD_440_ (comparison between background and non-fire year in Fig. 4). The volume concentrations of the coarse particles (V_C_) were 10 and 20 times higher than the background values. During the smoke episode, AE ~ 1.5 and AOD_440_ varied between 0.15 and 2.76, indicating the dominance of fine particles, such as smoke plumes (Fig. 4). Smoke observations showed a bimodal size distribution. As expected for biomass burning particles, the volume concentrations of the fine particles (V_F_) substantially increased with AOD_440_ (comparison between background and smoke in Fig. 4). The median radius for fine particles *r*_VF_ varied between 0.14 μm and 0.25 μm and agreed with reported biomass burning worldwide. Finally, aerosol conditions gradually returned to the previous state in the residual smoke episode.

### Sea surface particle changes

**Figure 5:**
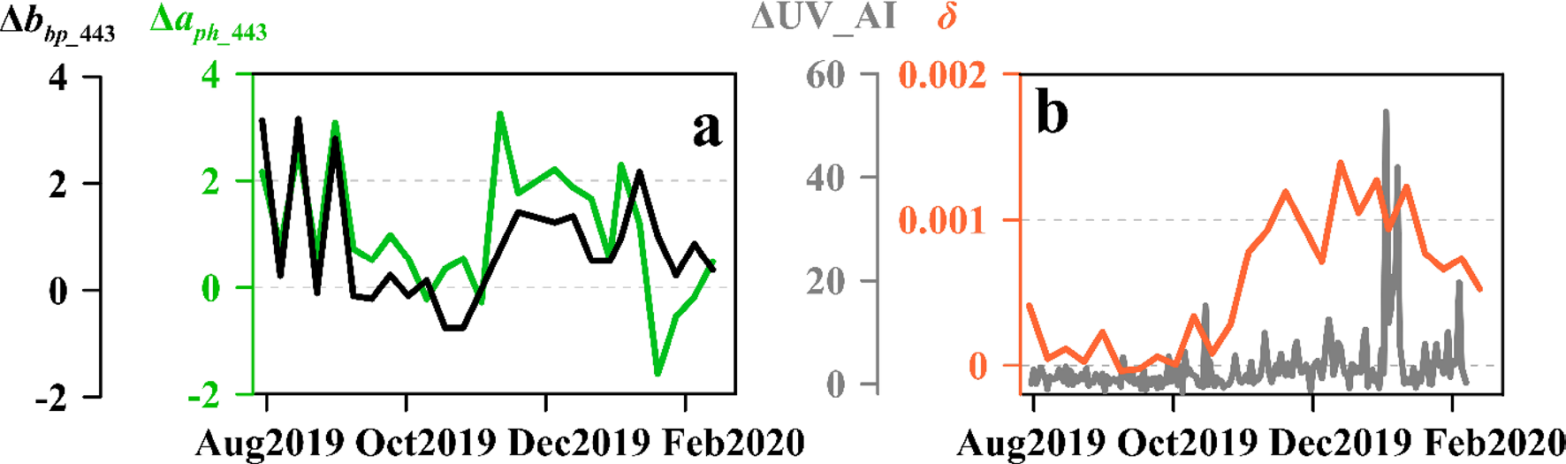
(**a**) *b*_*bp_*443_ and *a*_*ph_*443_ surface ocean anomalies in P1 between December 2019 and mid-February 2020, (**b**) residual scattering coefficient *δ* in surface water and ΔUVAI in P1.

Similar to the changes in atmospheric properties, the optical properties of the surface ocean were also greatly altered by atmospheric particles (Fig. 5). It illustrates that the 8-day standardized anomalies of particulate backscattering coefficients at 443 nm (Δ*b*_*bp*_443_) increased sharply on 17 November 2019 and fluctuated at 1 until the end of January 2020; thus, the content of total suspended particles was higher than the average level in the last 16 years. The standardized anomalies of absorption coefficient of phytoplankton at 443 nm (Δ*a*_*ph_*443_), a proxy for phytoplankton particulate matter, also showed a higher than average level on 9 November 2019 at 3.3 before gradually decreasing to the mean level. The variation in Δ*a*_*ph_*443_ indicates that the content of phytoplankton particles increased.

Figure 5b shows the residual scattering coefficients *δ* and standardized anomalies of UVAI (ΔUVAI). Before the arrival of the particles, the *δ* value was zero owing to the absence of atmospheric particle deposition; and the scattering coefficient was dominated by phytoplankton particles. *δ* reached 0.001 m^−1^ on 9 November 2019 and remained at approximately 0.0012 m^−1^. ΔUVAI increased significantly to 9.9 on 8 November 2019 and abruptly reached 52.6 on 31 December 2019. The time consistency between changes in optical proxies and bushfire emissions indicated that extraneous-source particle deposition altered the sea surface particulate compositions on the southeast coast of Australia.

### Response of phytoplankton biomass

**Figure 6:**
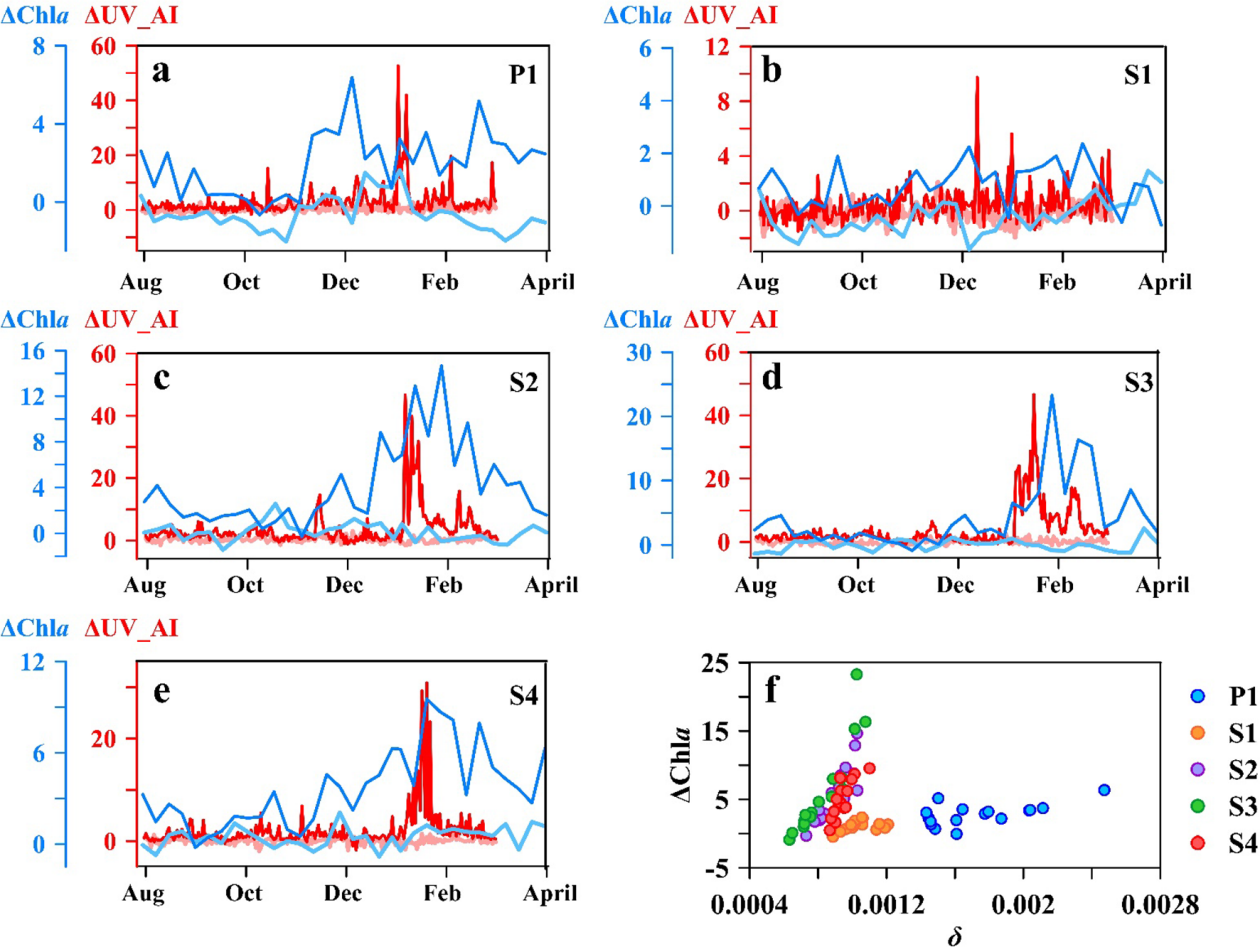
(**a**–**e**) ΔChl*a* and ΔUVAI data from P1 to S4. (**f**) relationship between *δ* and ΔChl*a* from 1 November 2019 to 26 February 2020. Light red and blue lines represented the ΔUVAI and ΔChla in the 2014–2015 non-fire season, respectively. Red and blue lines represented the ΔUVAI and ΔChla in the 2019–2020 bushfire season, respectively. Areas of interest from P1 to S4 are shown in Fig. 1.

Figure 6 shows the relationships between ΔUVAI and standardized anomalies of chlorophyll-a concentrations (ΔChl*a*) at five study sites from 28 July 2019 to 30 March 2020; the standardized anomalies of sea surface temperature (ΔSST) is included as the background for comparison. In the entire study area, no significant correlations were found between ΔSST and ΔChl*a* (*p* > 0.05), indicating that ΔSST was not a major factor of ΔChl*a*. And it is obvious that compared with the data in 2014–2015, there were significant impacts on both ΔUVAI and ΔChla in all study areas.

In P1, ΔChl*a* began increasing on 9 November 2019, peaked at 6.4 on 3 December 2019 (Fig. 6a), and decreased rapidly to average levels approximately one month after peaking. ΔUVAI fluctuated considerably on 8 and 22 November 2019 and rose rapidly to a maximum in early January 2020. After another peak on 1 February 2020, ΔUVAI in P1 quickly returned to normal levels. ΔChl*a* in S1 did not show significant changes (Fig. 6b) but increased slightly after the pollution event and peaked at 2.3 on 3 December 2019. ΔUVAI remained at high levels from 22 December 2019 to 3 January 2020 and peaked at 32.2 on 2 January.

The variations between ΔUVAI and ΔChl*a* in S2‒S4 were similar (Fig. 6c–e), with noticeable correlations between ΔChl*a* and ΔUVAI (S2: r = 0.48, *p* < 0.01; S3: r = 0.56, *p* < 0.01; S3: r = 0.62, *p* < 0.01) and a time lag of less than 8 days. The values of ΔChl*a* in S2‒S4 all increased in late November 2019, with maximum values 14.7, 23.3, and 9.6 in January; values did not return to average levels in S2‒S4 until the end of March 2020. A comparison of the variations of ΔUVAI and ΔChl*a* in the five study areas (Fig. 6a–e) revealed that atmospheric influence decreased from west to east (P1‒S4). Maximum ΔUVAI values in the smoke stage decreased from 52.6 to 30.8 in the easterly direction.

The relationship between *δ* and ΔChl*a* in the five areas is plotted in Fig. 6f. Except for a few outliers in the P1 and S1 areas, the particle deposition and the corresponding ΔChl*a* were well-correlated across the study area, showing an exponential increase with the *δ.* The slopes of the exponential relationship can be classified into two types: the lower slopes in P1 and S1 which were relatively close to fire source (r = 0.73, *p* < 0.001, N = 32) and the higher slopes in S2-S4 which were far from bushfire (r = 0.76, *p* < 0.001, N = 48).

### Response of biochemical properties

**Figure 7:**
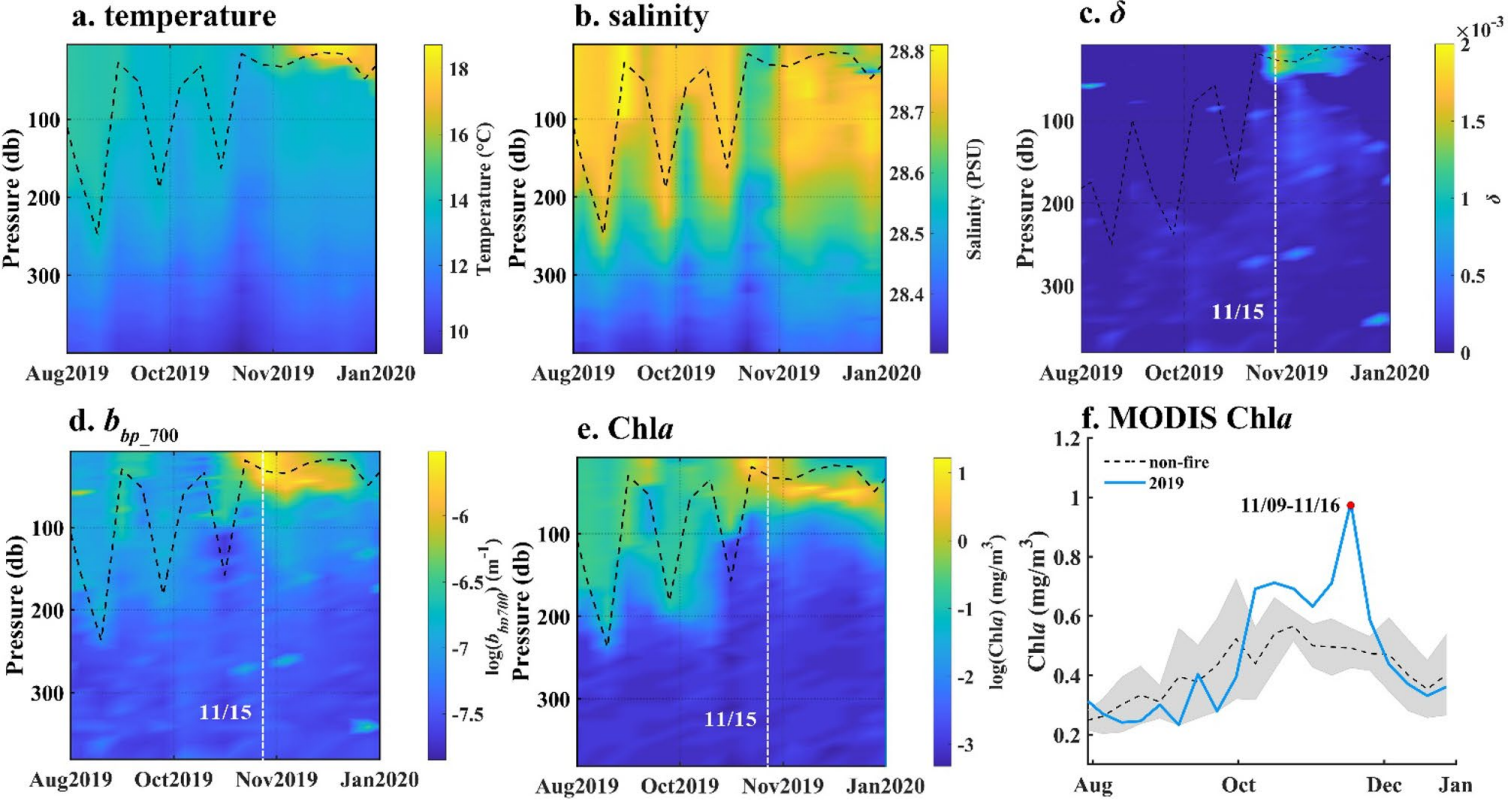
SOCCOM BGC-Argo float (No. 5905108) data and MODIS Aqua 8-day average Chl*a* in the same area. (**a**) temperature; (**b**) salinity; (**c**) *δ*; (**d**) *b*_*bp_*700_; (**e**) Chl*a*; (**f**) MODIS 8-day average Chl*a*. No. 5905108 float was located in the northeast sea of New Zealand, which is shown in Fig. 1b. The dashed black lines indicate the mixed layer depth (MLD). The white dashed lines indicate the date when surface Chl*a* reached its maximum.

To further explore the impact of particulate pollution on the biochemical properties of the seawater column, we analysed the biogeochemical Argo (BGC-Argo) data in the study area. Because buoy No. 5905108 (Fig. 7) was closer to the coast of Australia, the mineral dust stage had a greater impact on the upper water column. From 5 November 2019, Chl*a* in the water began to increase from the surface layer and peaked on 15 November. The average Chl*a* within 20 m of the surface layer was 2.45 mg/m^3^ (Fig. 7e). From August to October, particle optical backscatter at 700 nm (*b*_*bp*_700_) changed little throughout the water column; however, the concentration of surface particulates gradually increased from 5–15 November 2019 (Fig. 7d). After January 2020, the concentration of surface particulates decreased and returned to average levels. Figure 7c shows that the atmospheric inputs represented by *δ* were detected during this period from 15 November 2019 with *δ* at 0.001 m^−1^. Compared with BGC-Argo Chl*a* data, the MODIS Chl*a* in Fig. 7f showed the average Chl*a* on 9 to 16 November 2019 was extremely higher than the non-fire years average and mean + standard deviation.

**Figure 8:**
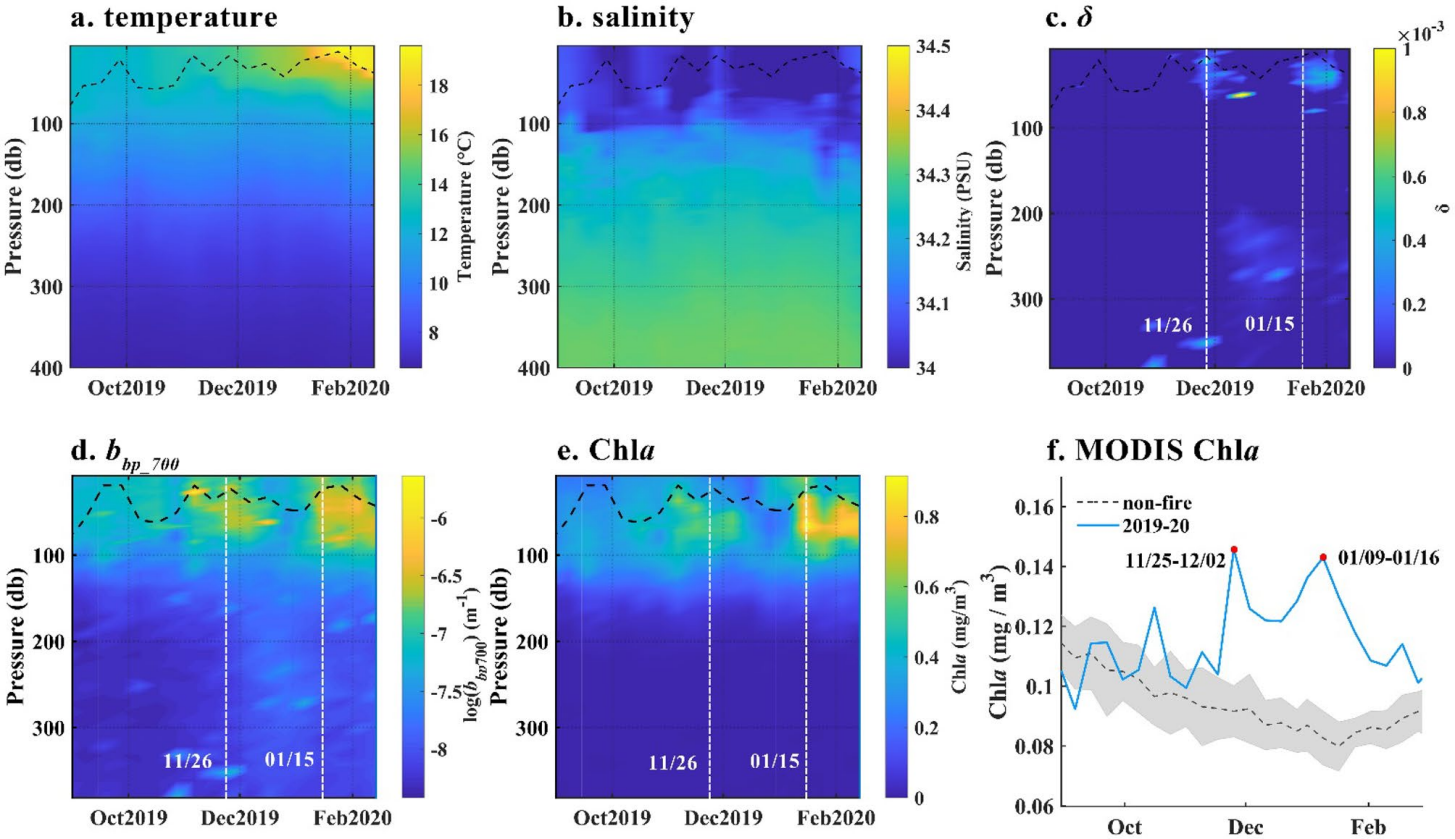
SOCCOM BGC-Argo float (No. 5904843) data and MODIS Aqua 8-day average Chl*a* in the same area. (**a**) temperature; (**b**) salinity; (**c**) *δ*; (**d**) *b*_*bp_*700_; (**e**) Chl*a*; (**f**) MODIS 8-day average Chl*a*. No. 5904843 float was located in the Southeast Pacific, which is shown in Fig. 1c. The dashed black lines indicate the MLD. The white dashed lines indicate the date when surface Chl*a* reached the maximum.

Similarly, for buoy No. 5904843 (Fig. 8) which was located in the Southeast Pacific, smoke particle deposition impacted Chl*a* and the surface particulate concentration; however, the impact was less than that of buoy No. 5905108. The smoke stage had a greater effect on the water column in the S4 region. Chl*a* in the surface water increased from 5 January 2020 and peaked at 1.85 mg/m^3^ at a depth of 64.7 m on 15 January 2020 (Fig. 8e); *b*_*bp*_700_ showed a similar pattern. Figure 8c showed that the atmospheric inputs represented by *δ* were detected on 26 November 2019 and 15 January 2020. In the meanwhile, the MODIS Chl*a* in Fig. 8f showed the average Chl*a* on 25 November to 2 December 2019 and 9 to 16 January 2020 reached peaks that were extremely higher than the non-fire years average and mean + standard deviation.

## Discussion

### Aerosol particle pollution and influence on the ocean

The particles released by the bushfires first affected the regional atmospheric conditions. Observations of different particle types, such as dust and smoke (“Temporal variation of aerosol types from AERONET”), suggested that the air particulate pollution derived from the 2019‒2020 Australian bushfires was complex. Convective updrafts in the combustion zone can carry large quantities of soil particles into the atmosphere^5,32^. The mobilised dust particles might be lifted by the updraft motion and mixed with the biomass burning aerosols^6^. Combining atmospheric particle size and composition with spatial variations (Figs. 2, 3, 4) revealed an impact concentrated primarily in the South Pacific marginal seas due to the dominance of coarse mineral dust particles and the potentially faster settling rate^33,34^. The fine smoke particles had a longer and more widespread influence, even across the South Pacific Ocean; this was consistent with the detailed backward trajectory analysis and TROPOMI observations^35,36^. According to the ΔUVAI in Fig. 6a–e, the particle load in the atmosphere decreased with increasing distance from the bushfire, perhaps due to continuous wet and dry deposition during atmospheric transport^34,37^.

The influence of aerosols on suspended particle composition in water is complex. On the one hand, the inorganic particle concentration increases due to particle deposition^38^. The responses of Δ*b*_*bp_*443_ and *δ*—which are the optical proxies of total particle concentration and exogenous particles, excluding the influence of phytoplankton—provided evidence obtained from remote sensing (Fig. 5) for the deposition of atmospheric particles on the sea surface. *δ* returned to zero by the end of January 2020 (Fig. 5b), likely due to supply disruption, dissolution, sinking of atmospheric particles, and rapid uptake by phytoplankton^39^.

The low correlation between ΔSST and ΔChl*a* in Fig. 6 indicates that ΔSST was not the primary factor of phytoplankton blooms in the oligotrophic area when compared to the input of exotic nutrients. Variations in ΔChl*a* in the five study areas (Fig. 6a–e) reveal that phytoplankton in both coastal Australia and the South Pacific Ocean responded to atmospheric particle deposition. The highest increase in Chl*a* was observed in P1 (0.45 mg/m^3^, Fig. 6a), indicating that coarse mineral dust was mostly settled in the P1 region, where potentially higher Fe inputs might cause an increase in phytoplankton biomass. In comparison, the relatively weak responses of ΔChl*a* to fine smoke particle deposition in the S1–S4 areas (Fig. 6b–e) were likely due to longer atmospheric transport distance and less particle deposition. On the contrary, the different slopes of the exponential relationship revealed that despite the distances of S2-S4 were far from the fires, little atmospheric deposition could lead to significant phytoplankton increases due to the extreme oligotrophic states. The utilisation of unit atmospheric deposition in P1 and S1 was lower compared to the hyper-oligotrophic areas.

The high correlation between aerosol particle deposition and the increase in ΔChl*a* (Fig. 6f) suggested that the uptake of extraneous nutrients led to the rapid growth of marine phytoplankton within 8 days; this was consistent with previous research^21,38^. However, the deviation points of P1 and S1, which are closer to the bushfire, indicated that ΔChl*a* did not respond to particle deposition, likely due to reduced Fe solubility for a shorter residence time of insoluble Fe^40^. The increases in the content of total suspended particles and phytoplankton observed in the water column at a maximum depth of 64.7 m (Figs. 7 and 8) agreed with in-situ observations^38^ and suggested a further influence on the biochemical and optical properties of the water column.

### Limitations and potential of this study

We acknowledge the limitations and potential of this study. The response of ΔChl*a* to particle deposition was not perfectly synchronous, as this study used 8-day average Chl*a* products. A more detailed time-lag analysis for the response of phytoplankton biomass to air particle deposition can be obtained by using a dataset with higher temporal resolution. The use of passive remote sensing for the high spatio-temporal resolution detection of atmospheric aerosol components and trace element monitoring for air pollution also presents limitations. In future work, the use of satellite-based lidar will facilitate the study and monitoring of atmospheric aerosol particle types and trace element pollution with high resolution and quality^35,41^.

The response of ΔChl*a* in our study showed the potential influence of bioavailable Fe deposition carried by different atmospheric components (Fig. 6). However, due to the lack of in-situ data for both atmospheric component analysis and iron contents in surface water, we were unable to obtain quantitative data of bioavailable iron in fire emissions. Therefore, further studies of the iron flux of mixed aerosols are necessary to help evaluate the impact of exogenous particles on marine ecology. We believe that the future deployment of additional Argo floats has the potential to provide more in-situ data on large-scale particle flux to fill the observation gap of the flux variation of particulate matter in the centre of the ocean.

As indicated by Harris and Lucas^42^, the long-term upward trend in fire weather in Australia is due primarily to anthropogenic activities and climate change (Supplementary Fig. 4a), which may increase the frequency and severity of drought and bushfire dust (Supplementary Fig. 4b) and the input of bushfire-derived particles and nutrients to the adjacent oceans. Although the 2019‒2020 wildfire season was episodic, this study indicated that Australian combustion-source particle pollution and its associated nutrition might have significant impacts on regional marine ecology.

## Conclusion

In this study, a comprehensive dataset (including AERONET, BGC-Argo, and multi-satellite observations) was used to study the spatio-temporal distribution of air particulate pollution during the 2019‒2020 Australian bushfires and their impacts on the ocean after particle deposition. Satellite-derived UVAI indicated that large-scale pollution lasted from the end of November 2019 until February 2020, leading to extensive optical and ecological impacts on the southeast coast of Australia and New Zealand, and across the South Pacific to South America.

Based on the analysis of atmospheric optical properties, four components of the aerosol pollution were identified, including background aerosols, dust particulate pollution dominated by coarse particles (more than 60%), wildfire smoke with a maximum AOD of 2.74, and residual subsidence. During the 2019–2020 bushfire season, both dust and smoke particles settled in the water and caused changes in the optical properties of water with *δ* reached 0.0012 m^−1^, which maintained for approximately 3 months.

The Chl*a* in both surface and water columns from coastal Australia to the South Pacific Ocean increased due to particle deposition and gradually decreased with the increase of distance from the location of bushfires for more than 3 months. ΔChl*a* in the Southeast Pacific Ocean reached a maximum value of 23.3 half of a month after the occurrence of atmospheric particle pollution. The high correlation between aerosol particle deposition and the increase in ΔChl*a* suggested rapid uptake of extraneous nutrients and growth of marine phytoplankton within 8 days; ΔChl*a* peaked at 6.4 in the Tasman Sea. In the water column, increases in the content of total particles and phytoplankton were observed with a maximum depth of 64.7 m. Such long-term continuous spatiotemporal remote sensing observations with extensive space coverage, in coordination with in-situ atmospheric and marine monitoring procedures, should be beneficial to further understand the bushfire pollution processes and detailed mechanisms of atmospheric deposition.

## Data and methods

The investigated regions of this study are illustrated in Fig. 1, which covers 20°S–45°S, 45°E–45°W, and most of the South Pacific Ocean. This section provides a detailed description of the datasets (including in-situ datasets), remote sensing observations, and methodology.

### AERONET data

Long-term and continuous aerosol optical, microphysical, and radiative properties were provided by the Aerosol Robotic Network (AERONET) website (https://aeronet.gsfc.nasa.gov). The atmospheric sounding station with a Cimel CE-318 Model T installed at the ‘Tumbarumba’ site (35.70830°S, 147.94990°E, 776.0 m, marked with the red star in Fig. 1) is operated by the Commonwealth Scientific and Industrial Research Organization. For this study, AERONET level 1.5 aerosol inversion data (Version 3) was used to show detailed in-situ particle characteristics of the fire emissions. The fine, coarse, and total modes of AOD_440_ and the AE were obtained from the spectral deconvolution algorithm (SDA)^43,44^. The volume particle size distribution (*dV(r)/dlnr*, µm^3^/µm^2^) were retrieved for 22 logarithmically equidistant discrete points (*r*_*i*_) and provided the percentage of aerosol particles with the observed aerosol equivalent spherical radius in the range of 0.05 µm ≤ *r* ≤ 15 µm^45^. More AERONET operational inversion algorithm information is described in detail by O'Neill*, *et al*.*^46^.

### Biogeochemical Argo data

The Argo project is an international program that observes the oceans from within using robotic profiling floats that drift with ocean currents^47^. The extensive array of biogeochemical Argo profiling floats are equipped with sensors collecting optical properties and biogeochemical parameters of the water^48–50^. Real-time float data were accessed online at ftp://ftp.ifremer.fr/ifremer/argo. The two floats (amol 5905108 and amol 5904843, Fig. 1b,c) selected for this study in S1 and S4 were deployed by the Southern Ocean Carbon and Climate Observations and Modelling project (SOCCOM) in 2017. This study considered temperature, salinity, Chl*a*, and *b*_*bp_*700_, which was used as a proxy for particle concentration from August 2019 to February 2020. Temperature and salinity profiles were used to compute the potential density of the seawater and to determine the depth of the mixed layer. Following de Boyer Montégut*, *et al*.*^51^, the mixed layer depth (MLD) was computed as the depth at which the change in potential density (from its value at 10 m, Δσ_θ_) exceeded 0.03 kg/m^3^.

### Satellite data

#### Optical properties of particles

The marine particulate optical constituents in this research refer to the composition of optically important marine particles in sea surface waters, specifically suspended particles, including marine phytoplankton and exogenous particulate matter. The optical properties of the particles are characterised by the particulate absorption and backscattering coefficients of light^52–54^. Moderate resolution imaging spectroradiometer (MODIS) Aqua ocean colour level-3 data, such as *b*_*bp_*443_ and *a*_*ph_*443_ calculated using the Generalised Inherent Optical Property (GIOP) model^55^, were selected for this study. *b*_*bp_*443_ and *a*_*ph_*443_ are determined by the concentration and physical properties of the total suspended particles and phytoplankton particle properties, respectively^52,54,56^. Level 3 mapped 8-day average data were freely downloaded from the NASA website (http://oceancolor.gsfc.nasa.gov/), with a resolution of 4 km from 1 January 2003 to 10 February 2020.

Phytoplankton and its appendants dominate the optical scattering properties with variations in *b*_*bp_*443_, which are highly correlated with Chl*a*^54,57^. Thus, the residual scattering coefficient *δ* (defined in Eq. (1)) is approximately zero^38^. Here, *δ* was used to describe the change in the sea surface particle composition, excluding the phytoplankton particle contribution and their appendants:

1 $$\delta { = }b_{bp\_443} - \alpha {(443)} \cdot {\text{Chl}}a^{\beta (443)}$$

where *α* (443) and *β* (443) are the regression coefficients^54^. The impacts induced by dust particles deposited from the dust storm could be clearly revealed by eliminating the contribution of phytoplankton to the water optical scattering coefficient^38^.

#### Sea surface temperature

The SST product was downloaded from the Group for High Resolution Sea Surface Temperature (GHRSST) website. The Global Australian Multi-Sensor SST Analysis (GAMSSA) Level-4 data (derived from Level-2 pre-processed products) are generated from multiple satellite datasets, including the Advanced Very High Resolution Radiometer, the Advanced Along Track Scanning Radiometer, and the Advanced Microwave Scanning Radiometer-EOS; in-situ data from ships; and drifting and moored buoys from the Global Telecommunications System^58,59^ using optimal interpolation^60,61^. In this study, daily SST data were obtained at https://www.ghrsst.org, with a resolution of 0.25° × 0.25° from 1 January 2003 to 10 February 2020.

#### Chlorophyll-*a* concentration

The chlorophyll-*a* concentration parameter, a proxy for phytoplankton biomass and ocean primary production, was used in this study to investigate the influence of pollution on biological and ecological systems in continental and open oceans^62,63^. The MODIS-Aqua 8-day average Chl*a* products with a spatial resolution of 4 km from 1 January 2003 to 31 March 2020 were downloaded from the NASA Ocean Color website (http://oceancolor.gsfc.nasa.gov/). The Chl*a* products were generated using the default chlorophyll algorithm, which employs the standard OC3/OC4 band ratio algorithm with the colour index from Hu*, *et al*.*^64^.

#### Aerosol index

The UVAI observations were derived from the Ozone Monitoring Instrument (OMI) onboard the Aura satellite, which is a contribution of the Netherlands’s Agency for Aerospace Programs in collaboration with the Finnish Meteorological Institute. Positive AI values generally represent absorbing aerosols (dust and smoke), whereas small or negative values represent non-absorbing aerosols and clouds^65^. Level-3 daily global total column ozone gridded products (OMTO3d) with a 1° × 1° resolution were obtained from the Giovanni platform (https://giovanni.gsfc.nasa.gov/giovanni/) from 1 January 2005 to 31 March 2020. Compared to the AOD retrieved from MODIS, UVAI inversion takes full advantage of the well-documented sensitivity of ultraviolet measurements to aerosol absorption^66^.

#### Active fire detection

The MODIS active fire products were used in this study between 2001 and 2020 for the analysis of the intensity of the bushfires. The global monthly fire location products (MCD14ML) contained the geographic location, date, and some additional information for each fire pixel detected by the Terra and Aqua MODIS sensors on a monthly basis. To ensure the quality of the product, the pixels with detection confidence over 70% were selected for the fire point detection in this study. The products are available from the University of Maryland fuoco SFTP server: fuoco.geog.umd.edu and detailed information can be found from the website https://modis-fire.umd.edu/af.html.

### Standardized anomaly calculations

To show the significant and important variations of the data, the standardized anomaly was calculated as the difference between the current period and the reference period and then divided by its reference period standard deviation. In this study, standardized anomalies (Δ*A*) for Chl*a*, *b*_*bp_*443_, and *a*_*ph_*443_ were calculated using Eq. (2) to evaluate the impact of pollution:

2 $${\Delta }A = (A - A_{mean} )/\sigma$$

where *A* is the 8-day observations of Chl*a*, *b*_*bp_*443_, and *a*_*ph_*443_ of each region between August 2019 and February 2020; *A*_*mean*_ is the average of Chl*a*, *b*_*bp_*443_, or *a*_*ph_*443_ calculated over the same period each year from 2003‒2018. *σ* is the standard deviations of annual Chl*a*, *b*_*bp_*443_, or *a*_*ph_*443_ in each region. Standardized anomalies of UVAI and SST were calculated using Eq. (2) as well, to remove the seasonal influence of UVAI and to exclude the abnormal influence of SST on Chl*a*, *b*_*bp_*443_, and *a*_*ph_*443_, respectively.

### Aerosol types identification

Aerosol classification from column-integrated observations is usually based on AOD_440_ and AE measurements^13,67^. Classification of background aerosols, mineral dust, and smoke is based on the method described by Gomez-Amo*, *et al*.*^13^, where AOD_440_ and AE are qualitative indicators of the air particle size. Aerosol types were classified by the values of AE in the presence of a significant increase in AOD. Therefore, for AE < 1, aerosol particle size distributions are mostly dominated by coarse models, such as sea salt and mineral dust. For AE > 1.5, fine particles associated with biomass burning emissions are dominant^68–70^. Mixed cases result for AE between 1 and 1.5, where the aerosol type cannot be identified^69^. The median radius for fine particles (r_VF_) varies between 0.12 and 0.25 of climatological values for biomass burning worldwide^67,70,71^.

It should be noted that this method was established on the statistical data. This method can only do the approximate classification of aerosol types by optical detection. If precise classifications of aerosol types are required, further analyses by biochemical methods are needed.

## Supplementary Information

Supplementary Information.
