# Supplementary material for: 2019‒2020 Australian bushfire air particulate pollution and impact on the South Pacific Ocean
Source: Sci Rep. 2021 Jun 10;11:12288. doi: 10.1038/s41598-021-91547-y (PMC8193010; doi:10.1038/s41598-021-91547-y)
Supplement: Supplementary file 1 — Supplementary Information. [file 41598_2021_91547_MOESM1_ESM.pptx]

## Slide 1
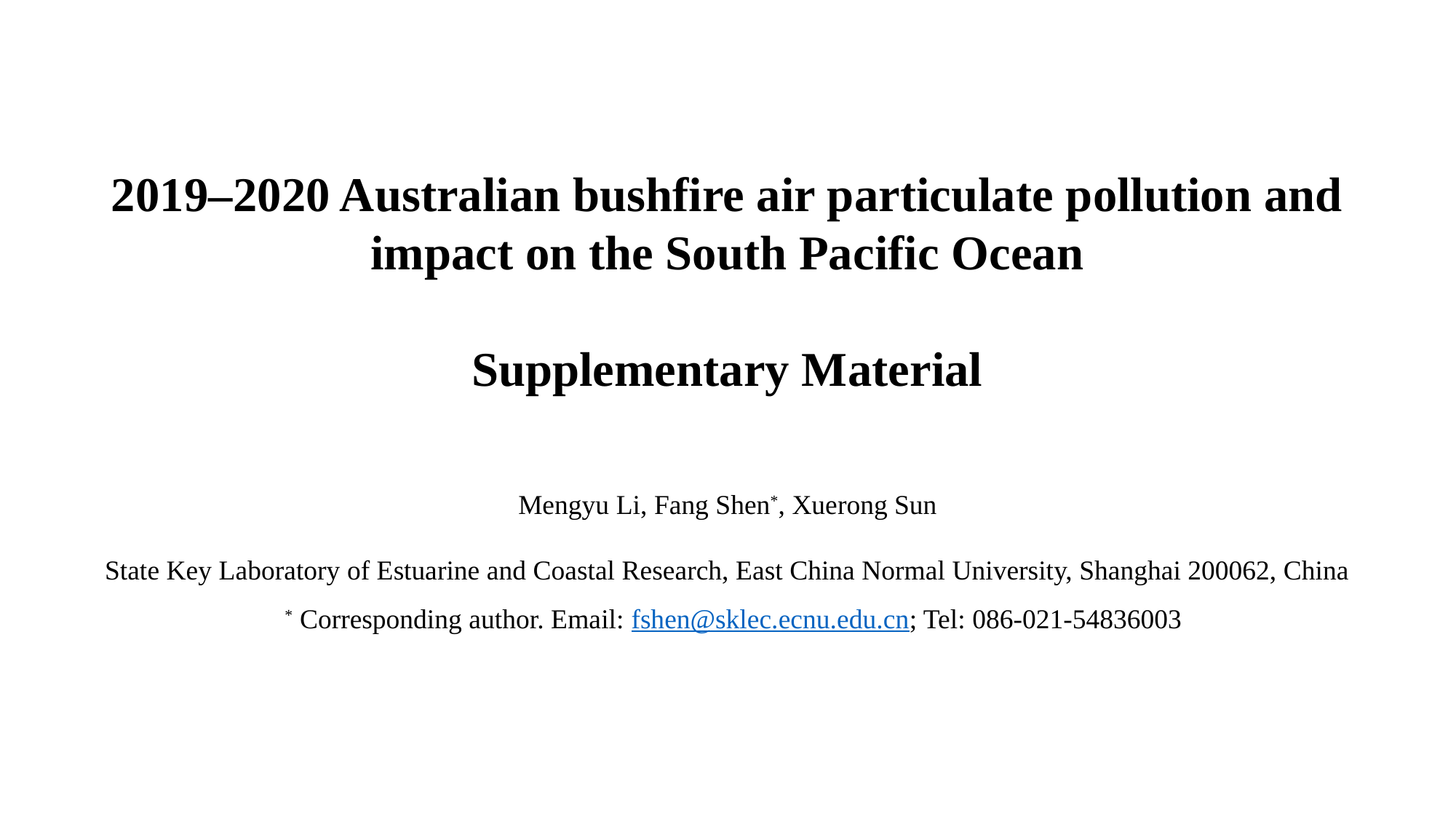

2019‒2020 Australian bushfire air particulate pollution and impact on the South Pacific Ocean
Supplementary Material
Mengyu Li, Fang Shen*, Xuerong Sun
State Key Laboratory of Estuarine and Coastal Research, East China Normal University, Shanghai 200062, China
* Corresponding author. Email: fshen@sklec.ecnu.edu.cn; Tel: 086-021-54836003

## Slide 2
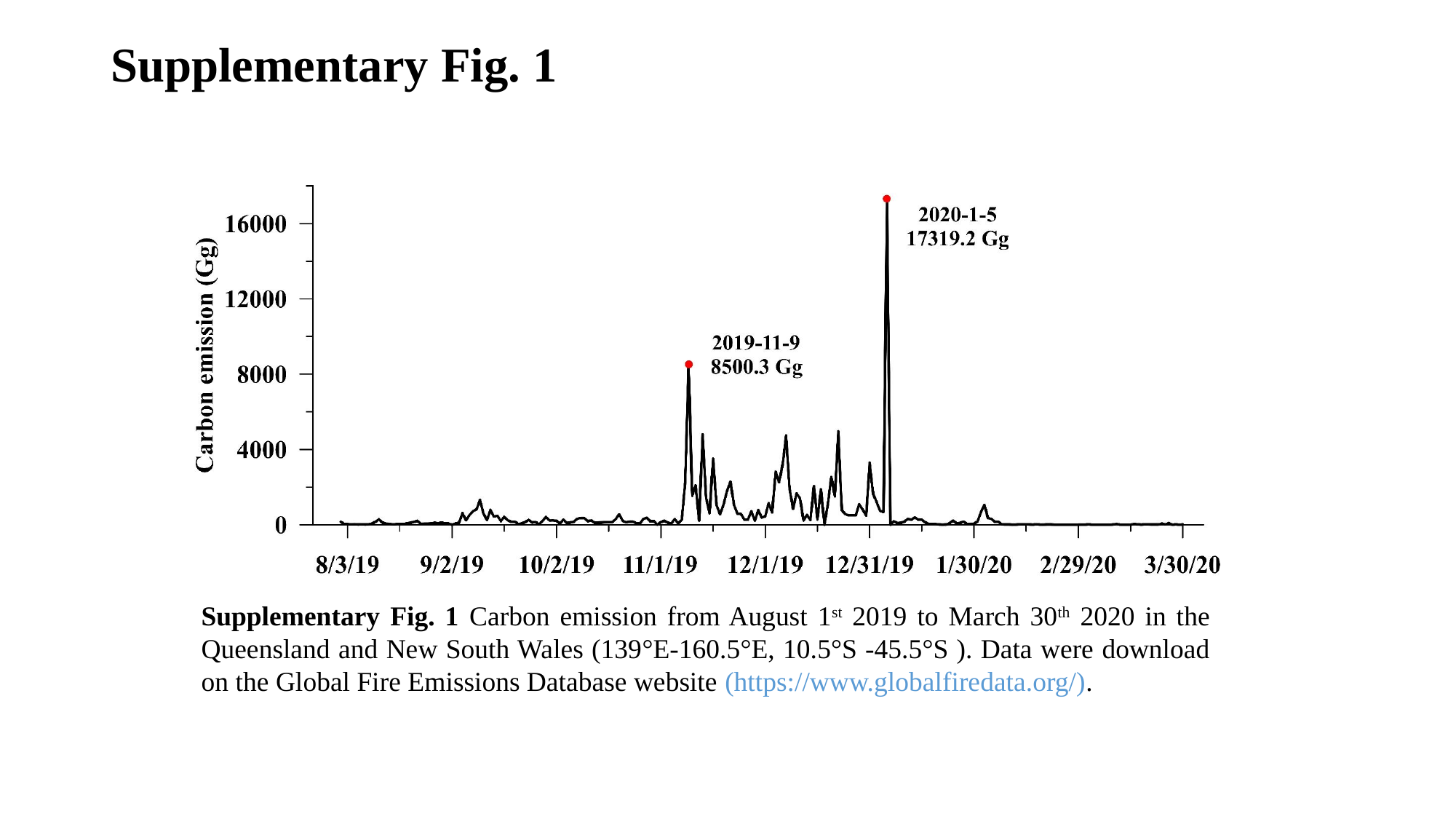

# Supplementary Fig. 1
Supplementary Fig. 1 Carbon emission from August 1st 2019 to March 30th 2020 in the Queensland and New South Wales (139°E-160.5°E, 10.5°S -45.5°S ). Data were download on the Global Fire Emissions Database website (https://www.globalfiredata.org/).

## Slide 3
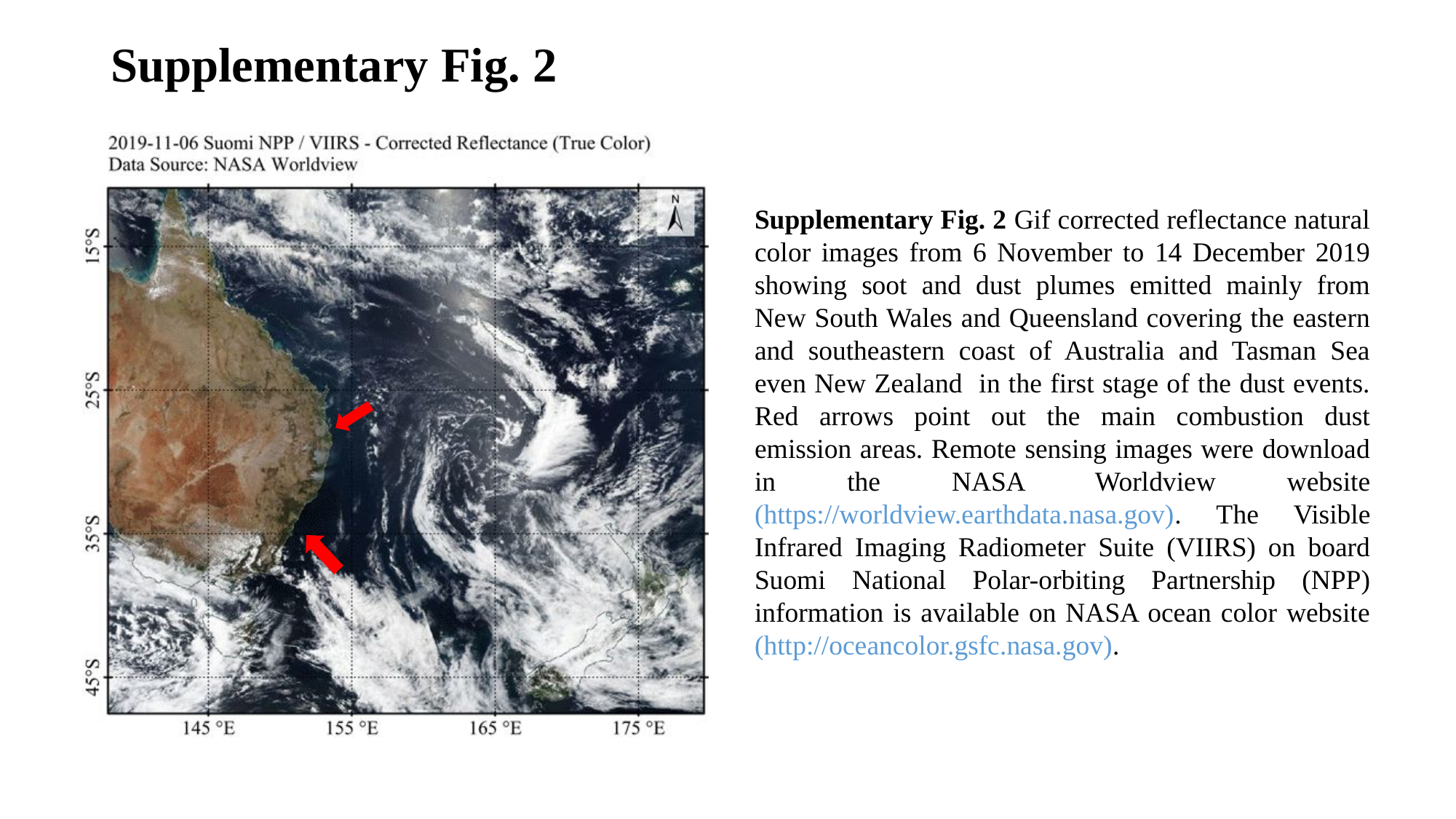

# Supplementary Fig. 2
Supplementary Fig. 2 Gif corrected reflectance natural color images from 6 November to 14 December 2019 showing soot and dust plumes emitted mainly from New South Wales and Queensland covering the eastern and southeastern coast of Australia and Tasman Sea even New Zealand in the first stage of the dust events. Red arrows point out the main combustion dust emission areas. Remote sensing images were download in the NASA Worldview website (https://worldview.earthdata.nasa.gov). The Visible Infrared Imaging Radiometer Suite (VIIRS) on board Suomi National Polar-orbiting Partnership (NPP) information is available on NASA ocean color website (http://oceancolor.gsfc.nasa.gov).

## Slide 4
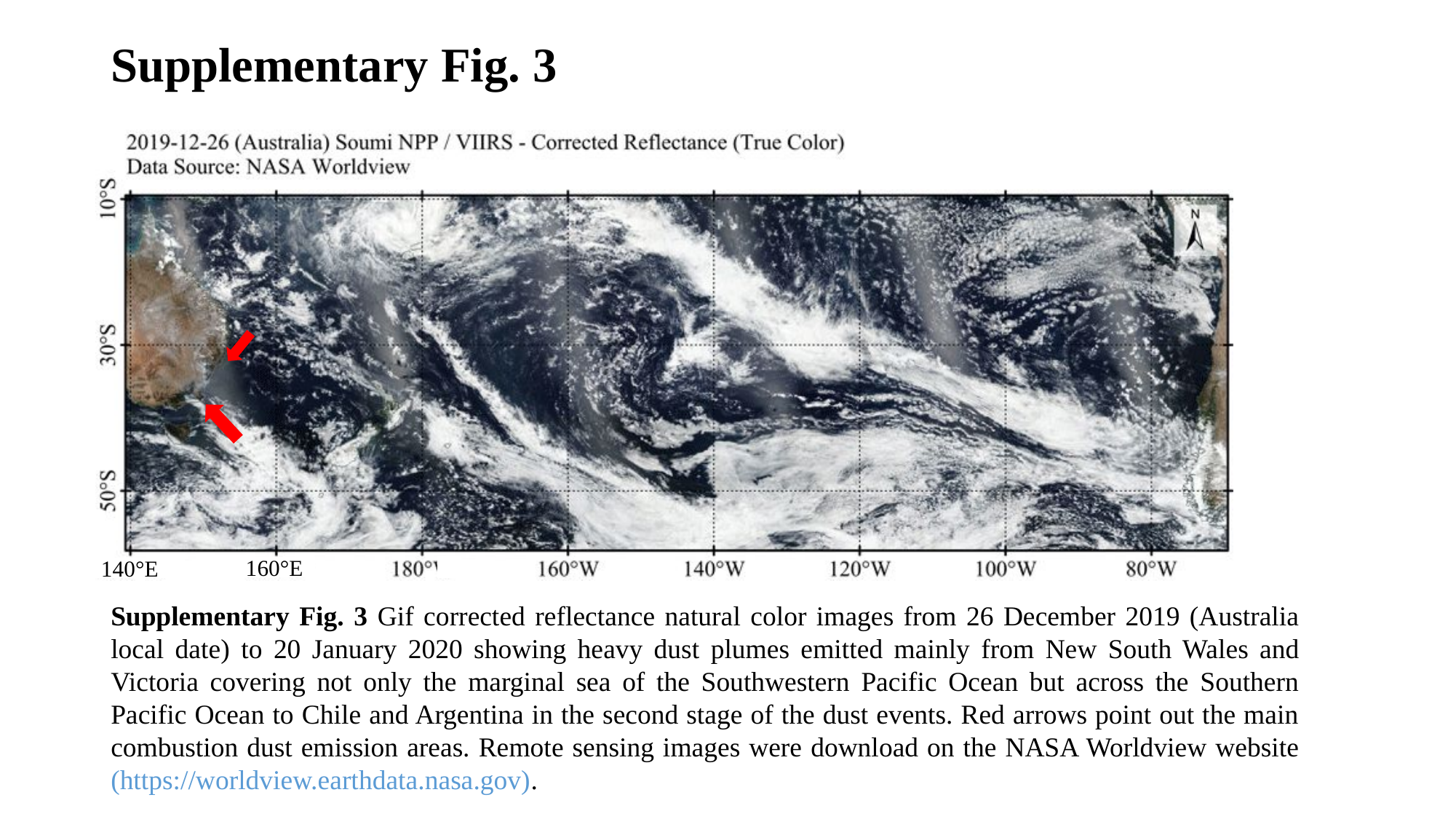

# Supplementary Fig. 3
160°E
140°E
Supplementary Fig. 3 Gif corrected reflectance natural color images from 26 December 2019 (Australia local date) to 20 January 2020 showing heavy dust plumes emitted mainly from New South Wales and Victoria covering not only the marginal sea of the Southwestern Pacific Ocean but across the Southern Pacific Ocean to Chile and Argentina in the second stage of the dust events. Red arrows point out the main combustion dust emission areas. Remote sensing images were download on the NASA Worldview website (https://worldview.earthdata.nasa.gov).

## Slide 5
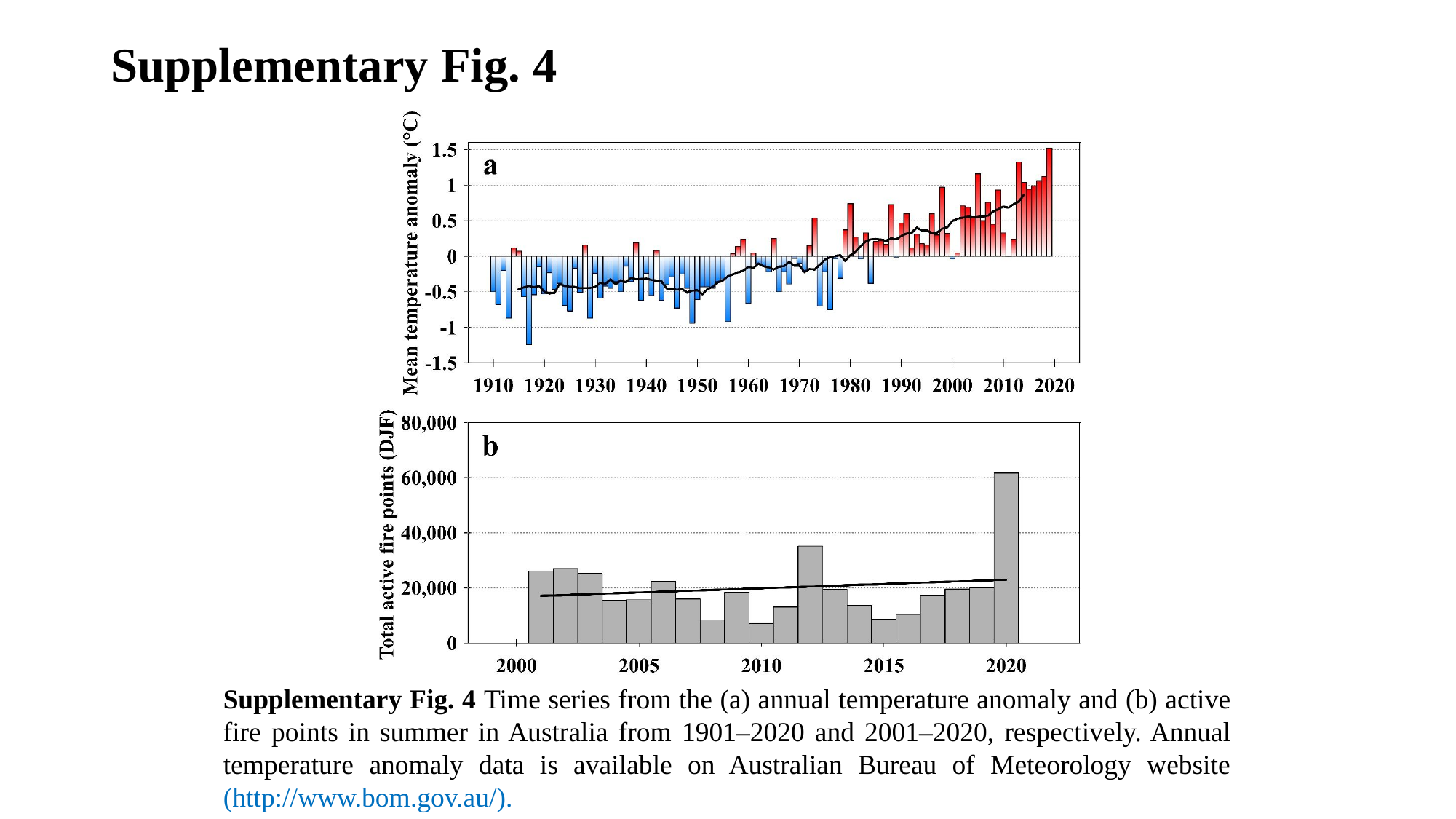

# Supplementary Fig. 4
Supplementary Fig. 4 Time series from the (a) annual temperature anomaly and (b) active fire points in summer in Australia from 1901–2020 and 2001‒2020, respectively. Annual temperature anomaly data is available on Australian Bureau of Meteorology website (http://www.bom.gov.au/).
